# An internet traffic classification method based on echo state network and improved salp swarm algorithm

**DOI:** 10.7717/peerj-cs.860

**Published:** 2022-02-28

**Authors:** Meijia Zhang, Wenwen Sun, Jie Tian, Xiyuan Zheng, Shaopeng Guan

**Affiliations:** 1 School of Data Science and Computer Science, Shandong Women’s University, Jinan, Shandong, China; 2 School of Information and Electronic Engineering, Shandong Technology and Business University, Yantai, Shandong, China

**Keywords:** Internet traffic, Classification, Echo state network, Salp swarm algorithm, Hyperparameter optimization

## Abstract

Internet traffic classification is fundamental to network monitoring, service quality and security. In this paper, we propose an internet traffic classification method based on the Echo State Network (ESN). To enhance the identification performance, we improve the Salp Swarm Algorithm (SSA) to optimize the ESN. At first, Tent mapping with reversal learning, polynomial operator and dynamic mutation strategy are introduced to improve the SSA, which enhances its optimization performance. Then, the advanced SSA are utilized to optimize the hyperparameters of the ESN, including the size of the reservoir, sparse degree, spectral radius and input scale. Finally, the optimized ESN is adopted to classify Internet traffic. The simulation results show that the proposed ESN-based method performs much better than other traditional machine learning algorithms in terms of per-class metrics and overall accuracy.

## Introduction

With the expansion of the Internet scale, the increasing kinds of network applications and huge amount of network traffic restrict the effective management of network equipment. Network security and congestion problems have become increasingly prominent. Internet traffic classification is the foundation of network security and management. According to different network application classes, the traffic generated by network communication is classified, which can optimize network configuration, reduce the risks of network security, and provide better service based on analysis of user behavior (Callado et al., 2009).

Traditional internet traffic classification schemes mainly comprise port-based approach and deep packet inspection-based approach (Zhang et al., 2014). According to the port mapping table stipulated by the Internet Assigned Numbers Authority (IANA), the port-based approach classifies the network traffic of a specific port into the corresponding network application (Aceto et al., 2010). However, with the popularity of novel network applications such as FTP and P2P, lots of random ports are used to transfer data. Hence, the port-based approach is rapidly eliminated  (Rezaei & Liu, 2019). Since emerging services dynamically bind ports, the deep packet inspection (DPI)-based approach has become a relatively mature technology recognized in the industry. This method identifies the application by analyzing the protocol signature in the payload (Finsterbusch et al., 2013). Nevertheless, due to the increase of the links in the backbone network, analyzing the complete payload not only has large computational overhead, but also may cause unnecessary privacy disputes. In addition, the payload encryption technology limits the identification capability of the DPI-based approach.

Machine learning (ML) does not rely on parsing protocol content or matching protocol port to classify network application, but distinguishes network applications according to different statistical features in the transmission process of the traffic generated by different applications. It is not affected by dynamic ports, load encryption or network addresses (Nguyen & Armitage, 2008; Pacheco et al., 2018). At present, the ML-based methods include Support Vector Machine (SVM) (Yuan et al., 2010; Este, Gringoli & Salgarelli, 2009; Sun et al., 2018; Jing et al., 2011; Cao et al., 2017), Decision Tree (Tong, Qu & Prasanna, 2017; Li & Moore, 2007), K-Nearest Neighbors (KNN) (Qi et al., 2020; Ma, Du & Cao, 2020), Bayes (Dias et al., 2019; Zhang et al., 2012; Moore & Zuev, 2005), and Neural Network, *etc.* Among them, Neural Network processes information by simulating the thinking mode of human brain and has the characteristics such as self-organization, self-learning and self-adaptation. It is applied to network traffic classification and has achieved satisfactory classification performance (Pacheco et al., 2018; Liu et al., 2017). Liu et al. (2020) proposed an HTTP traffic classification method based on the bidirectional Gated Recurrent Unit (GRU) Neural Network. This method utilized the bidirectional GRU to extract the forward and backward features of byte sequences in the session, and then employed the attention mechanism to assign the weight of the features according to their contributions. Wang et al. (2017) cut the original data traffic and input it into the Convolutional Neural Network (CNN) to classify network traffic. Yang et al. (2018) adopted CNN to extract high-dimensional features of the network traffic, and then extracted the representative features from these features based on the AutoEncoder (AE), which achieves network traffic identification. Zeng et al. (2019) proposed a framework of the network traffic classification based on Deep-Full-Range (DFR), which respectively utilizes CNN, LSTM and Stacked Auto Encoder (SAE) to extract the spatial, temporal and coding features of the original traffic, and combines these features to achieve comprehensive understanding of the original traffic. Lotfollahi et al. (2020) used header information and payload data to train CNN and SAE respectively. The experiment shows that the identification performance of CNN is slightly better than that of SAE. Ren, Gu & Wei (2021) proposed a tree structural recurrent neural network (Tree-RNN), which divides a large classification into small classifications by using the tree structure. A specific classifier is set for each small classification after division. With multiple classifiers employed, Tree-RNN can sovle the problem of relatively poor classification effect for multi-classification. Li et al. (2021) proposed a method of mobile service traffic classification based on joint deep learning with attention mechanism. In the first step, a joint deep learning model is designed as a basic classifier, which learns features of mobile service traffic from multiple time scales. In the second step, the attention mechanism is adopted to aggregates the basic predictions generated in the first step, which filters out useless information. Compared with traditional ML algorithm, the neural network-based methods have achieved better identification effect. However, the methods have some disadvantages such as long training time and high computational cost since the structure of adopted neural network is complex and their weights have to be determined through multiple iterations. With respect to the related researches, they are summarized in Table 1, including the simple descriptions on approach and comments.

**Table 1:** Summary of related work.

Work	Simple description	Comments
Aceto et al. (2010)	Classify the network traffic of a specific port into the corresponding network application	Be affected by dynamic ports
Finsterbusch et al. (2013)	Identify the application by analyzing the protocol signature in the payload	Have large computational overhead, and may cause unnecessary privacy disputes
Liu et al. (2020)	Utilize the bidirectional GRU to extract the forward and backward features of byte sequences in the session, and then employed the attention mechanism to assign the weight of the features according to their contributions	Have long training time and high computational cost
Wang et al. (2017)	Cut the original data traffic and input it into the CNN to classify network traffic	
Yang et al. (2018)	Adopt CNN to extract high-dimensional features of the network traffic, and then extract the representative features from these features based on the AE	
Zeng et al. (2019)	Utilize CNN, LSTM and SAE to extract the spatial, temporal and coding features of the original traffic, and combine these features to achieve comprehensive understanding of the original traffic	
Lotfollahi et al. (2020)	Use header information and payload data to train CNN and SAE respectively	
Ren, Gu & Wei (2021)	Divide a large classification into small classifications by using the tree structure	
Li et al. (2021)	Design a joint deep learning model as a basic classifier, and then adopt the attention mechanism to aggregates the basic predictions generated in the first step	
This paper	Utilize the advanced SSA to optimize the hyperparameters of the ESN, and then adopt the optimized ESN to classify Internet traffic	Simplify the training process, and have the characteristics of easy implementation and fast training speed

Echo state network (ESN), as a kind of neural network, adopts the reserve pool composed of sparsely connected neurons as the hidden layer to perform high-dimensional and non-linear representation of the input data (Grigoryeva & Ortega, 2018). It only needs to train the weights from the reserve pool to the output layer, which simplifies the training process and solves problems of traditional neural networks such as complex training and difficult determination of network structure. ESN, with the characteristics of easy implementation and fast training speed, has good application prospects in time series forecasting (Wang et al., 2018; Long, Zhang & Li, 2019; Zhang et al., 2019; Duan & Wang, 2015). In this paper, we propose an internet traffic classification method based on ESN.

Although the echo state network has advantages in solving the above problems, there are also some problems. For example, the stability of the reserve pool will affect the generalization ability of the network, which is easy to cause problems such as over-fitting. Hu et al. proposed an improved method of ESN, which combines ESN and deep learning, and used the efficient learning ability of deep learning to improve the stability of the reserve pool (Hu, Wang & Lv, 2020). When ESN is used to classify network traffic, hyperparameters such as the input scale and sparse degree are of great importance to the classification performance. The results obtained through different hyperparameter configurations vary greatly. Therefore, selecting the optimal hyperparameters are critical to the results of network traffic classification. The traditional hyperparameter optimization method finds out the hyperparameters of the ESN by manual setting. However, this method has the disadvantage of time-consuming and it is difficult to select the optimal hyperparameters. Swarm Intelligence (SI) Optimization algorithms are inspired by the collective behavior of creatures. Since the individuals in the population can interact and share information, the SI algorithm has the characteristics of strong flexibility and fast convergence, and can provide satisfactory solutions when applied to the automatic optimization of hyperparameters. At present, the Genetic Algorithm (GA)  (Zhong et al., 2017), the Particle Swarm Algorithm (PSO) (Chouikhi et al., 2017), the Fruit Fly Optimization Algorithm (FOA) (Tian, 2020; Zhang et al., 2020), the Differential Evolution Algorithm (DE) (Hu, Wang & Tao, 2021) and the Grey Wolf Optimizer (GWO) (Kohli & Arora, 2018) algorithm have been adopted to automatically optimize hyperparameters of ESN. However, the reservoir of ESN contains many nodes and its search space is large, hence the above-mentioned algorithms are not suitable for optimizing hyperparameters with a large range of values. Salp Swarm Algorithm (SSA) is a new type of SI algorithm that simulates the foraging behavior of salps in the biological world. It guides search optimization by simulating the population behavior of salps sailing and foraging in ocean, and has the advantages of high optimization accuracy, strong search ability and good robustness. SSA has been employed to function optimization (Rizk-Allah et al., 2019), combination optimization (Abualigah et al., 2020), and shortest path solution (Ateya et al., 2019), *etc.*, and can obtain the optimal solution. Mirjalili et al. (2017) evaluated SSA on 19 well-known mathematical functions and 2 optimization problems, and compared it with state-of-art SI algorithms such as PSO, GA and FOA. The results show that SSA has better optimization performance than other SI algorithms. However, like other SI algorithms, SSA also has some shortcomings, such as inadequate spatial search at the early stage and reduced population diversity at the iterative process. To enable SSA to find the optimal solution more accurately, we introduce Tent mapping with reverse learning, polynomial operator and dynamic mutation strategy to improve SSA, and then adopt the advanced SSA to optimize the important hyperparameters of ESN automatically. Our main contributions are as follows:

(1) Network traffic classification plays a vital role in analyzing user behavior, enhancing network controllability, improving service quality and ensuring network security. Considering ESN has the advantages such as excellent classification performance, fast training speed and easy implementation, we propose a classification method of Internet traffic based on ESN.

(2) We improve the SSA. Firstly, Tent mapping with reverse learning is introduced to initialize the population, which makes the distribution of the initial population position uniform and improves the search efficiency of the SSA. Secondly, polynomial operator is used to maximize the diversification of search domain and improve the global exploration ability of the SSA. Finally, a dynamic mutation strategy is adopted to increase the population diversity at the later stage and avoid the SSA falling into local optimum.

(3) Hyperparameters of ESN such as input scale and sparse degree are of great importance to the classification performance. The reservoir of ESN contains many nodes and large search space, thus the optimization effect of the traditional hyperparameter optimization method still needs to be improved. We use the advanced SSA to optimize important hyperparameters of ESN.

The rest of the paper is organized as follows: ‘Materials & Methods’ introduces the ESN and discusses its important hyperparameters. Then, we improves the SSA and describes the network traffic classification method. In ‘Results’, we perform experiments to verify the effectiveness of the proposed scheme. Finally, we summarizes our work in ‘Conclusion’.

## Materials & Methods

### ESN

**Figure 1:**
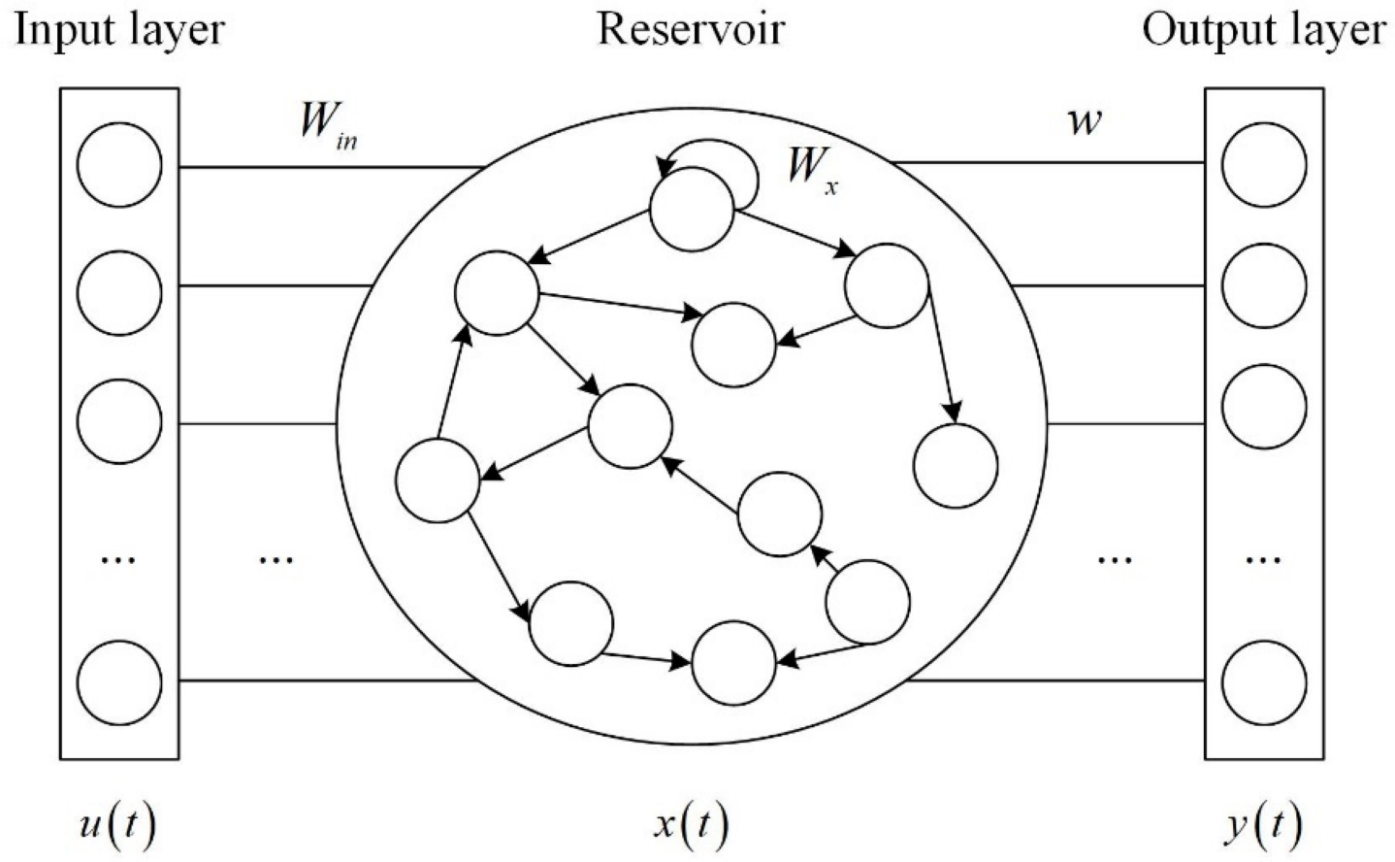
ESN structure.

ESN is composed of input layer, reservoir and output layer. The reservoir contains hundreds of sparsely connected neurons, and the connection weights between neurons are randomly generated and fixed. Figure 1 shows the ESN structure.

The state equation and output equation of ESN are as follows (Han & Xu, 2017):

(1) \begin{eqnarray*}x(t)& =\varphi \left( {W}_{in}u(t)+{W}_{x}x(t-1)+{W}_{back}y \left( t-1 \right) \right) \end{eqnarray*}

(2) \begin{eqnarray*}y(t)& ={f}_{out} \left( {W}_{out} \left( u \left( t \right) ,x \left( t \right) ,y \left( t-1 \right) \right) \right) .\end{eqnarray*}

where *u*(*t*) ∈ *R*^*M*×1^ is the input vector, *y*(*t*) ∈ *R*^*M*×1^ is the output vector, *b*_*x*_ ∈ *R*^*N*×1^ is the input bias, and *b* ∈ *R*^*M*×1^ is the output bias. The state *x*(*t*) ∈ *R*^*N*×1^ at the current time is calculated from the input vector *u*(*t*) at the current time *t* and the state of the reservoir *x*(*t* − 1) at the previous time *t* − 1. *φ*(⋅) is the activation function of the neuron, which can select Sigmoid function or tanh function. The element of the input-reservoir connection weight matrix *W*_*in*_ ∈ *R*^*N*×*K*^ is in the interval [−1,1]. *W*_*x*_ ∈ *R*^*N*×*N*^ is the internal connection weight matrix of the reservoir. *W*_*back*_ ∈ *R*^*N*×*L*^ is the output-reservoir connection weight matrix. *W*_*out*_ ∈ *R*^*L*×(*K*+*N*×*L*)^ is the output connection weight matrix. *W*_*in*_, *W*_*x*_ and *W*_*back*_ are generated randomly and remain unchanged during the training phase of ESN. The network only needs to train the output connection weight matrix *W*_*out*_, which reduces the computational complexity.

The core of ESN is the reservoir. Its performance depends on four crucial hyperparameters: the size of the reservoir *N*, spectral radius *R*, sparse degree *D* and input scale *S*. How to select these hyperparameters is very important (Duan & Wang, 2015).

(1) Size of the reservoir

The size of the reservoir *N*, as the most important hyperparameter affecting the performance of ESN, is the number of neurons in the reservoir. The larger the number of neurons is, the better the classification performance is. However, if the number of neurons is too large, overfitting will be caused.

(2) Spectral radius

The spectral radius *R* is the absolute value of the maximum eigenvalue of the internal connection weight matrix *W*_*x*_ of the reservoir. *R* < 1 is a necessary condition to ensure the stability of the network.

(3) Sparse degree

The sparse degree *D* indicates the sparsity of neuron connections. The neurons in the reservoir are sparsely connected rather than fully connected. The larger the value is, the stronger the nonlinear approximation ability is.

(4) Input scale

The input scale *S* refers to the scale factor before data is input into the reservoir, and represents the range of input connection weight. According to Eq. (1), it determines the working interval of activation function and the extent to which the input data affect the state of the reservoir. It’s usually in the interval [0,1].

The performance of ESN relies heavily on the above hyperparameters, and the results obtained through different hyperparameter configurations vary greatly.

### The advanced SSA

SSA is a new type of SI algorithm with the advantages of high optimization accuracy, good robustness and high convergence rate. SSA divides the population into leaders and followers. They form a salp chain to perform population optimization. To enable it to find the optimal solution more accurately, we improved the SSA.

### Population initialization

The uniform distribution of the population can effectively maintain the population diversity and improve the optimization performance. The initial population of SSA is generated randomly. Lack of prior knowledge leads to uneven distribution and poor initial population diversity.

To enhance population diversity and search efficiency, the Tent mapping with reverse learning is introduced to initialize the population. The Tent mapping has the characteristics of randomness, ergodicity and regularity. Thus, it can generate initial salp population with rich diversity. Then, the reverse learning strategy is adopted to optimize the population and generate the reverse population. At last, the population generated by the Tent mapping and its reverse population are merged and sorted. The salps with better fitness value are selected to form the initial population. Tent mapping with reverse learning is introduced to expand the search range of the population, reduce invalid search, and improve the search efficiency. The population generated by Tent mapping is expressed as follows (Arora & An, 2019):

(3) \begin{eqnarray*}{x}_{d+1}= \left\{ \begin{array}{@{}l@{}} \displaystyle 2{x}_{d},0\leq {x}_{d}\leq \frac{1}{2} \\ \displaystyle 2 \left( 1-{x}_{d} \right) , \frac{1}{2} \leq {x}_{d}\leq 1 \end{array} \right. \end{eqnarray*}

where *x*_*d*_ and *x*_*d*+1_ are the respective values of the *d*th and (*d* + 1)th dimensions of the population generated by the Tent mapping.

The reverse learning strategy is used for the population generated by Tent mapping, and the obtained reverse population is as follows:

(4) \begin{eqnarray*}{x}_{d+1}^{{^{\prime}}}={u}_{d}+{l}_{d}-{x}_{d+1}.\end{eqnarray*}

where ${x}_{d+1}^{{^{\prime}}}$ are the values of the (*d* + 1)th dimension of the reverse population; *u*_*d*_ is the upper limit of the *d*th dimension; and *l*_*d*_ is the lower limit of the *d*th dimension.

### Leader position update

Adequate global exploration is helpful for the algorithm to obtain better optimization results. Traditional SSA conducts global exploration by introducing random numbers into leader position update. However, the introduced random numbers have strong randomness and cannot fully perform global exploration. Polynomial operator can maximize the diversification of search domain and enhance the convergence speed of the SSA at the later stage. Therefore, we introduce polynomial operator into leader position update.

The formula of improved leader position update is as follows:

(5) \begin{eqnarray*}{X}_{d}^{{l}^{{^{\prime}}}}& ={F}_{d}+\delta \cdot \left( {u}_{d}-{l}_{d} \right) \end{eqnarray*}

(6) \begin{eqnarray*}\delta & = \left\{ \begin{array}{@{}l@{}} \displaystyle { \left[ 2u+ \left( 1-2u \right) { \left( 1-{\delta }_{1} \right) }^{{\eta }_{m}+1} \right] }^{ \frac{1}{{\eta }_{m}+1} -1}, u\leq 0.5\\ \displaystyle 1-{ \left[ 2 \left( 1-u \right) +2 \left( u-0.5 \right) { \left( 1-{\delta }_{2} \right) }^{{\eta }_{m}+1} \right] }^{ \frac{1}{{\eta }_{m}+1} }, u\gt 0.5 \end{array} \right. \end{eqnarray*}

(7) \begin{eqnarray*}{\delta }_{1}& = \left( {X}_{d}^{l}-{l}_{d} \right) / \left( {u}_{d}-{l}_{d} \right) \end{eqnarray*}

(8) \begin{eqnarray*}{\delta }_{2}& = \left( {u}_{d}-{X}_{d}^{l} \right) / \left( {u}_{d}-{l}_{d} \right) .\end{eqnarray*}

where *F*_*d*_ is the food position (*i.e.,* the optimal position of salp in the population); *u* is the random number in the interval [0,1]; *η*^*m*^ represents the distribution index; ${X}_{d}^{{l}^{{^{\prime}}}}$ is the position of updated leader on the *d*th dimension; and ${X}_{d}^{l}$ is the position of current leader on the *d*th dimension.

### Follower position update

At the later stage of iteration, followers will gather near the current food source, which reduces the population diversity and the search ability of the SSA. To avoid the premature phenomenon at the later stage, we introduce dynamic mutation strategy into the follower position update, which increases the diversity of salp population at the later stage and improves the convergence accuracy of the SSA. At present, researchers have proposed a variety of mutation algorithms, such as Gaussian mutation and Cauchy mutation (Li et al., 2017). Compared with Gaussian operator, Cauchy operator has longer wings and can generate a large range of random numbers, so that the SSA has a greater chance to avoid local optimum. In addition, less time is needed to search the nearby area when the peak value is low. Therefore, we introduce Cauchy mutation into follower position update.

The formula of improved follower position update is as follows:

(9) \begin{eqnarray*}{X}_{d}^{{m}^{{^{\prime}}}}& = \frac{1}{2} \left( {X}_{d}^{m}+{X}_{d}^{m-1} \right) +\eta \ast C \left( 0,1 \right) \left( {u}_{d}-{l}_{d} \right) \end{eqnarray*}

(10) \begin{eqnarray*}\eta & ={e}^{-\lambda \frac{t}{T} }.\end{eqnarray*}

where ${X}_{d}^{m}$ and ${X}_{d}^{m-1}$ are the respective positions of the *m*th and (*m* − 1)^th^ followers on the *d*pth dimension before the update; ${X}_{d}^{{m}^{{^{\prime}}}}$ is the positions of the *m*th followers on the *d*th dimension after the update; *η* is the mutation weight which decreases with the increase of the number of iteration; *T* is the maximum number of iteration; *t* is the current number of iteration; *λ* is a constant and its value is 10; *C*(0, 1) is a random number generated by the Cauchy operator and its scaling parameter is 1.

If the mutation frequently occurs during the iteration process, it will not be conducive to the algorithm convergence. Therefore, the mutation trigger mechanism is introduced. If the fitness value of mutated follower position is better, the Cauchy mutation will be introduced into the follower position. Otherwise, the Cauchy mutation will not be introduced.

The steps of the advanced SSA (ASSA) are shown in Algorithm 1.

**Table:** 

Algorithm 1: ASSA.
Input: population size *NP*, maximum number of iteration *T*
Output: the optimal position of salp in the population
1 Adopt the Tent mapping with reverse learning to initialize the salp population.
2 for *t* = 1 to *T* do
3 Calculate the fitness value of salps in the population
4 Sort salps in the population according to fitness value
5 Choose food. The salp position with the best fitness is regarded as the food position
6 Choose leaders and followers. After selecting the food, there are *N* − 1 salps remaining in the population. The salps with the first half of the fitness value are regarded as the leaders, and the others are regarded as followers
7 Update the leader position according to formula Eq. (5)
8 Update the follower position according to formula Eq. (9)
9 end for

In Algorithm 1, we at first employ the Tent mapping with reverse learning to initialize the salp population. Then, we calculate the fitness values of salps in the population and sort salps in the population according to the fitness values. The salp position with the best fitness value is regarded as food position. After the food position is selected, there are *N* − 1 salps left in the population. The salps with the first half of the fitness values are regarded as the leaders, and the others are regarded as followers. We update the position of leaders and followers respectively according to Eqs. (5) and (9). Repeat the above steps until meeting the stopping condition (*e.g.,* the maximum number of iterations). Finally, output the salp position with the best fitness value.

### Performance analysis of ASSA

**Figure 2:**
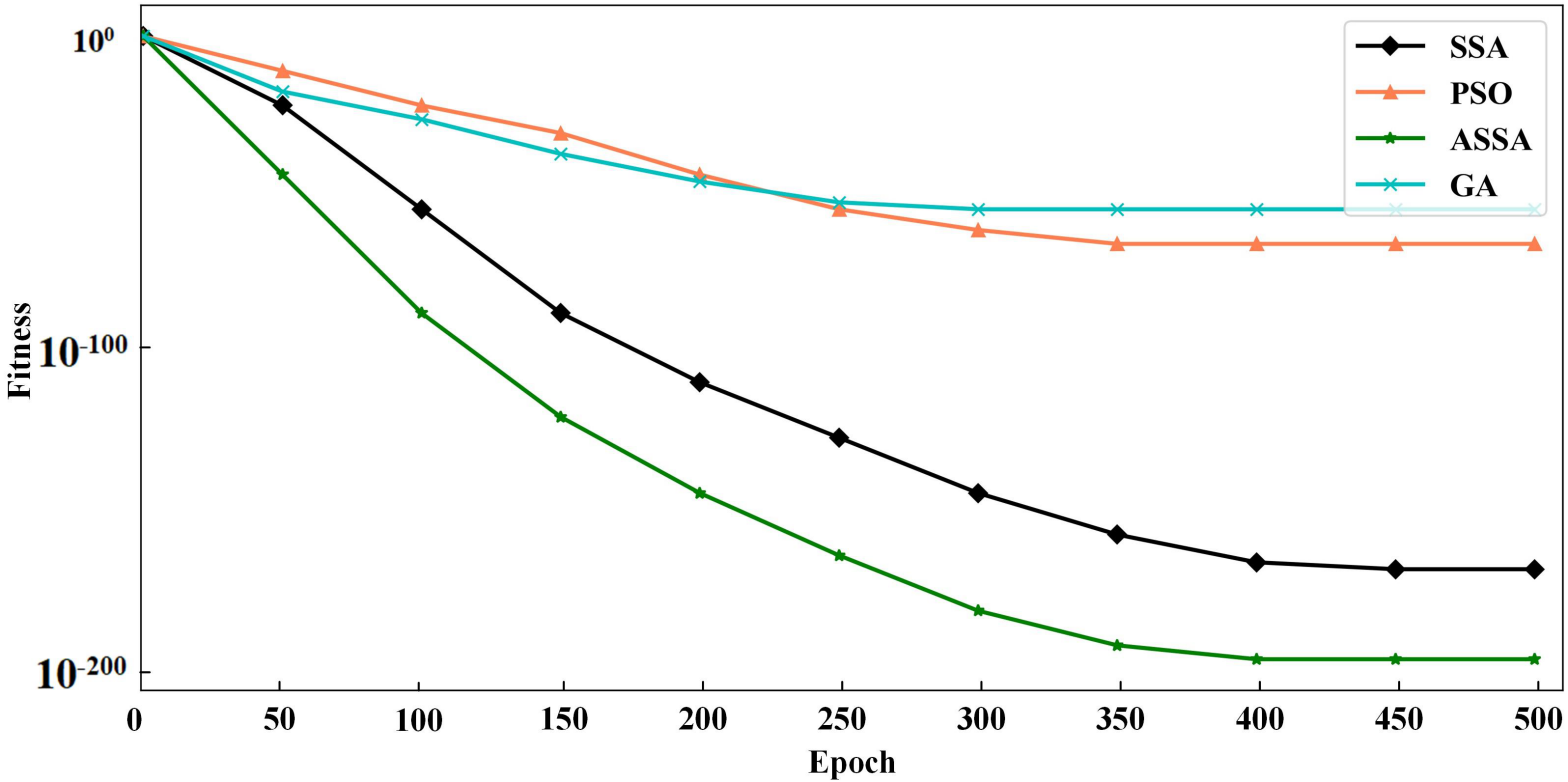
The testing results with Sphere function.

**Figure 3:**
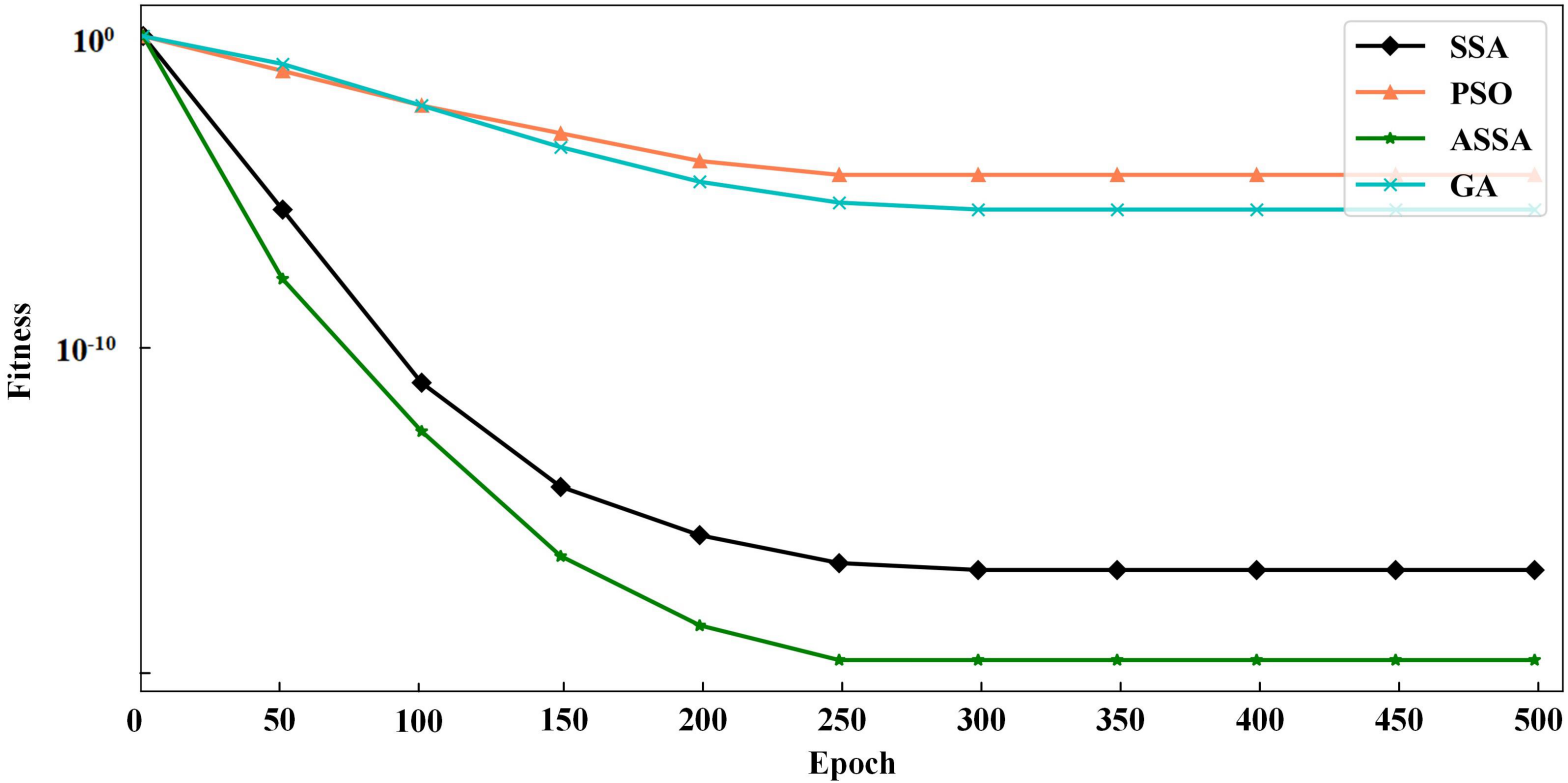
The testing results with Griewank function.

To verify the performance of the ASSA algorithm, two typical functions of Sphere and Griewank, are selected for function optimization and convergence test of the algorithm. Sphere is a unimodal function and Griewank is a multimodal function. We compare ASSA with the PSO, GA and SSA. The number of algorithm iterations is set to 500. The testing results of the four algorithms on the functions are shown in Figs. 2 and 3.

It can be seen from Figs. 2 and 3 that ASSA has obvious advantages in convergence speed and accuracy compared to the PSO, GA, and SSA. Therefore, the performance of the ASSA is significantly improved over the traditional optimization algorithms.

### Scheme design

**Figure 4:**
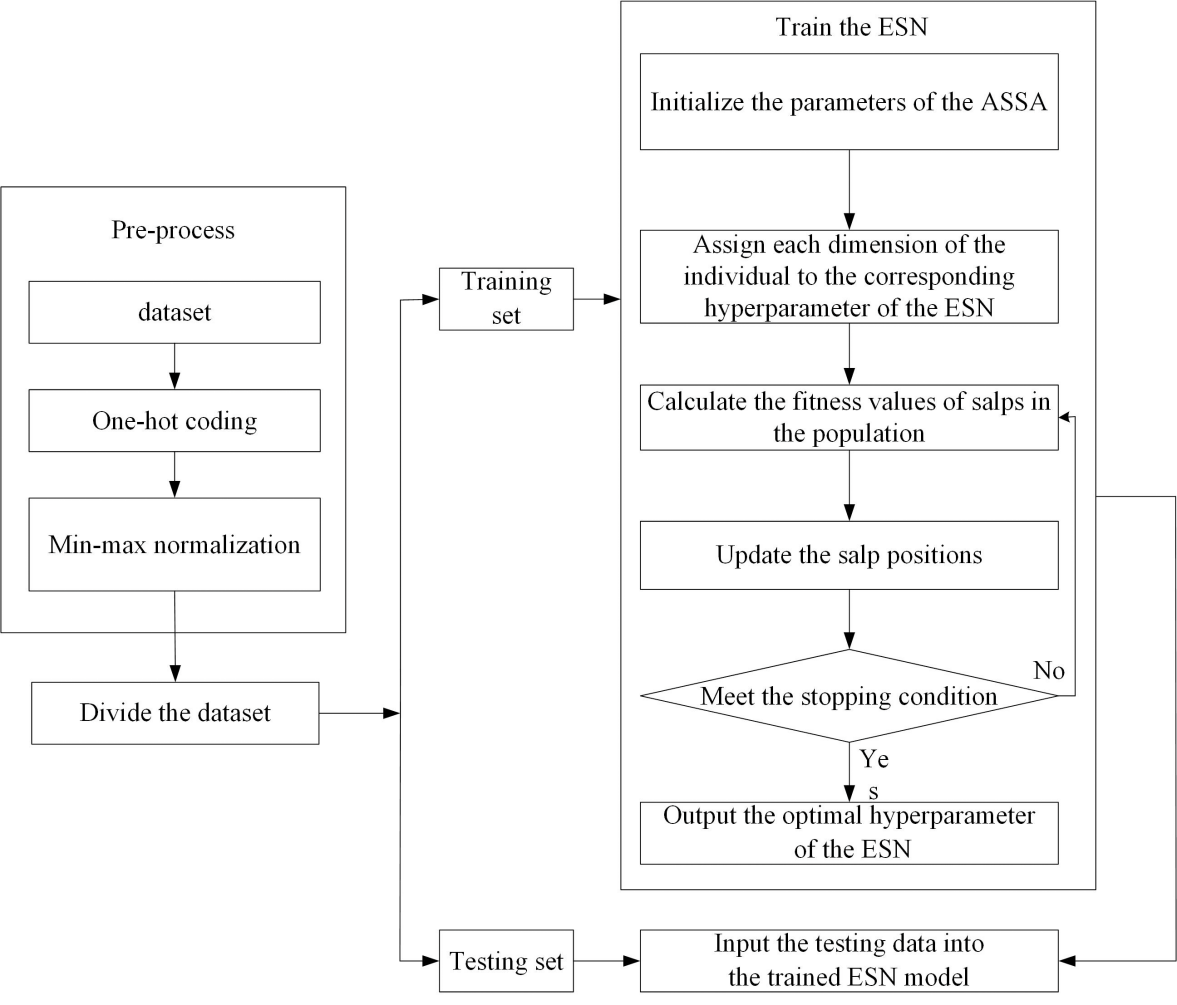
The flowchart of ESN-based network traffic classification.

We use ASSA-optimized ESN for Internet traffic classification. The basic idea is as follows: The ASSA is utilized to find the salp position with the best fitness value. At the end of iteration, each dimension of the salp position is assigned to the corresponding hyperparameter of the reservoir of ESN, which establishes the network traffic classification model. The flowchart of ESN-based network traffic classification is shown in Fig. 4. The steps of ESN-based network traffic classification are as follows:

**Step 1:** Pre-process the network traffic classification dataset. There are two pre-processing ways.

(1) One-hot coding. We implement one-hot coding for discrete features.

(2) Min-max normalization. The large difference between the data of the same attribute affects the training of the network. Therefore, we perform min-max normalization on continuous features.

**Step 2:** Divide the dataset into training set and testing set.

**Step 3:** Train the ESN and adjust its hyperparameters.

(1) Initialize the parameters of the ASSA, such as the salp population size and the maximum number of iterations. Use the Tent mapping with reverse learning to initialize the salp population. Each dimension of the individual in the population represents a hyperparameter of the ESN, and different hyperparameters have different ranges. Therefore, each dimension of the individual is constrained.

(2) Assign each dimension of the individual to the corresponding hyperparameter of the ESN: the size of the reservoir *N*, spectral radius *R*, sparse degree *D* and input scale *S*.

(3) Calculate the fitness values of salps in the population according to the training samples and fitness function, and arrange the fitness values in ascending order to find the salp position with the optimal fitness value. If the stopping condition is met, go to **Step 4**. Otherwise, go to **Step 3**. The fitness function is the overall accuracy of network traffic classification.

**Step 4:** Input the testing data into the trained ESN model, and then get the classification result of each sample.

## Results

### Experimental dataset

To verify the effectiveness of the proposed scheme, we conduct experiments using two public datasets called Moore dataset (Lopez-Martin et al., 2017) and NISM dataset (Demertzis & Iliadis, 2016), which are from raw traffic data. With a long interval between them, they have different data terminals and IP addresses, which enables the effective evaluation of the generality of the proposed scheme. Each dataset includes the training set and the testing set. The proportion of each category in the training set and testing set is consistent with that of the original dataset. 100,000 samples are randomly selected as the testing set, and the others are the training set.

(1) Moore dataset

The Moore dataset comes from the traffic flowing through the network outlet of a biological institute from 0 to 24 h on August 20, 2003. 377,526 network samples are obtained from the 24 h traffic by sampling algorithm. They are divided into 12 application classes. Each sample contains 249 attributes, among which the last one is the category corresponding to each sample. Table 2 reports the Moore dataset statistics.

**Table 2:** Moore dataset statistics.

**Sample type**	**The number of training samples**	**The number of testing samples**	**Proportion**
WWW	241,186	86,906	86.906%
Mail	21,000	7,567	7.567%
Ftp-data	4,261	1,536	1.536%
Ftp-pasv	1,976	712	0.712%
Ftp-control	2,245	809	0.809%
Services	1,543	556	0.556%
Database	1,947	701	0.701%
P2P	1,539	555	0.555%
Attack	1,318	475	0.475%
Mutimedia	423	153	0.153%
Interactive	82	28	0.028%
Games	6	2	0.002%
Totality	277,526	100,000	100%

(2) NISM dataset

The NISM dataset comes from the network traffic of the Information Technology Operations Center of the U.S. Military Academy in 2013. The dataset contains 713,851 network traffic samples, which are divided into 11 application classes. The NISM dataset statistics are shown in Table 3.

**Table 3:** NISM dataset statistics.

**Sample type**	**The number of training samples**	**The number of testing samples**	**Proportion**
DNS	32,691	5,325	5.325%
Ftp	1,486	242	0.242%
Http	10,236	1,668	1.668%
Telent	1,076	175	0.175%
Lime	555,738	90,533	90.533%
Local forwarding	2,199	358	0.358%
Remote forwarding	2,083	339	0.339%
Scp	2,102	342	0.342%
Sftp	2,074	338	0.338%
Shell	2,142	349	0.349%
X11	2,025	330	0.330%
Totality	613,851	100,000	100%

### Evaluation index

We use the following evaluation indexes to evaluate the classification performance. The samples in the training set are divided into *m* application classes. *TP*_*i*_ represents the number of its samples that are correctly classified as belonging to class *i*. *FN*_*i*_ represents the number of its samples that are misjudged as other types. *FP*_*i*_ represents the number of the samples from other application classes that are misjudged as belonging to class *i*. The evaluation indexes are defined as follows.

(1) The accuracy rate of class *i*

(11) \begin{eqnarray*}Ac{c}_{i}=T{P}_{i}/ \left( T{P}_{i}+F{N}_{i} \right) .\end{eqnarray*}

(2) The recall rate of class *i*

(12) \begin{eqnarray*}{R}_{i}=T{P}_{i}/ \left( T{P}_{i}+F{N}_{i} \right) .\end{eqnarray*}

(3) The F-measure of class *i*

(13) \begin{eqnarray*}{F}_{i}=2\times {P}_{i}\times {R}_{i}/ \left( {P}_{i}+{R}_{i}. \right) \end{eqnarray*}

(4) The overall accuracy rate

(14) \begin{eqnarray*}OA=\sum _{i=1}^{m}T{P}_{i}/\sum _{i=1}^{m} \left( T{P}_{i}+F{P}_{i} \right) .\end{eqnarray*}

Among the above indexes, the accuracy and recall rate of per class can reflect the classification performance of the proposed scheme for per class. The F-measure, as the harmonic average of the accuracy and the recall rate, gives a better comprehensive evaluation of the classification ability. In addition, the overall accuracy can reflect the proportion of correctly classified samples to all samples.

### Experimental results

(1) Experiments of ESN hyperparameter optimization

The range of ESN hyperparameters is set as follows: The size range of the reservoir is set as [30, 300], the range of spectral radius [0.1, 0.99], the range of sparse degree [0.01, 1], and the range of input scale [0.1, 1]. Mirjalili et al. evaluated SSA on more than 20 test functions and 5 optimization problems, and compared them with typical swarm intelligence optimization algorithms such as PSO, GA, and ABC  (Rizk-Allah et al., 2019). The experimental results show that, compared with above algorithms, SSA has better optimization performance. Therefore, referring to the literature (Hu, Wang & Tao, 2021), the population size is set as 21, and the maximum number of iterations 20. On the Moore dataset and the NISM dataset respectively, the optimal hyperparameter values of ESN selected by ASSA are shown in Table 4.

**Table 4:** Hyperparameters of ESN.

**Dataset**	**Hyperparameter**	**Value**
Moore dataset	the size of the reservoir	98
	spectral radius	0.78
	sparse degree	0.89
	input scale	0.95
NISM dataset	the size of the reservoir	77
	spectral radius	0.83
	sparse degree	0.78
	input scale	0.90

**Figure 5:**
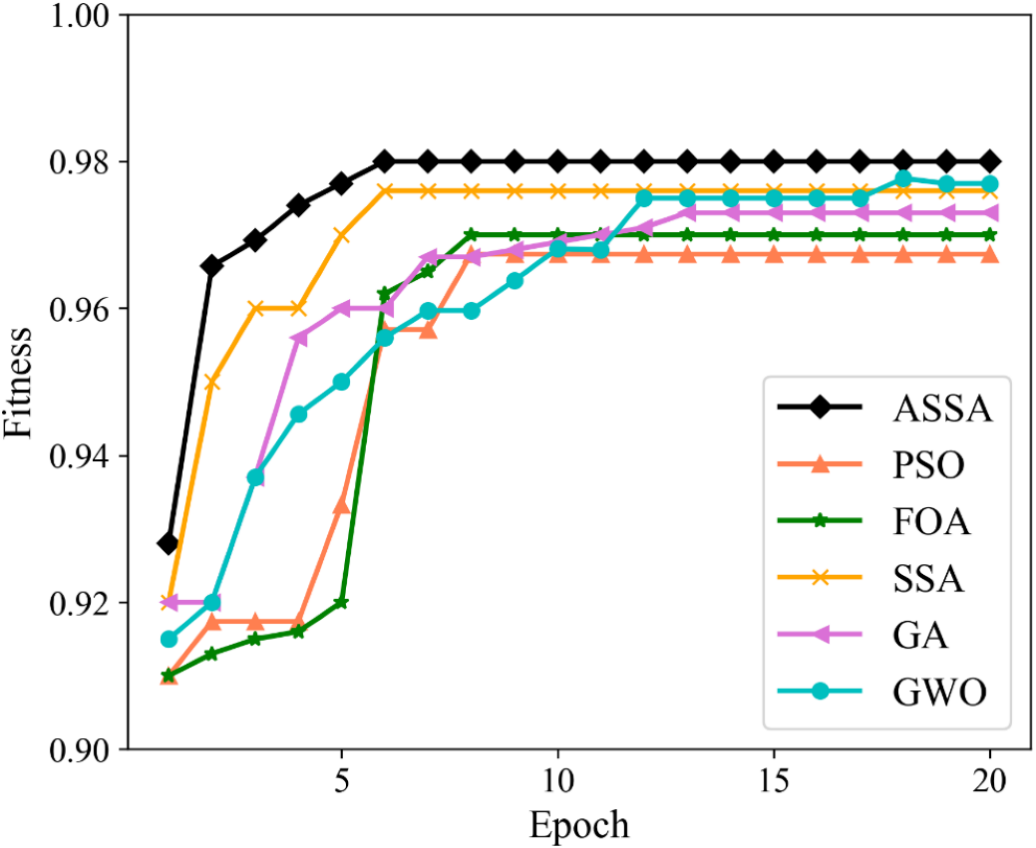
The changing curves of fitness values on the Moore dataset.

**Figure 6:**
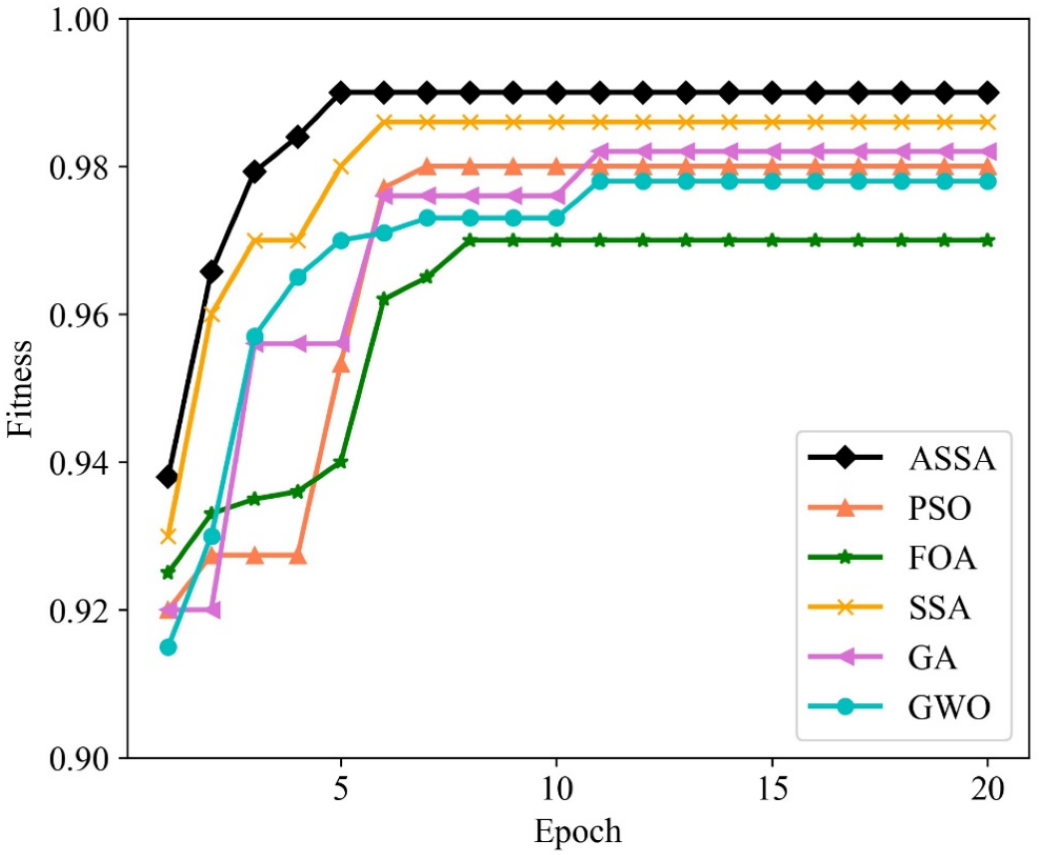
The changing curves of fitness values on the NISM dataset.

We utilize PSO, FOA, GA, GWO, SSA and ASSA to optimize the hyperparameters of the ESN. The fitness function, with a direct impact on the optimal solution of the model, is usually defined by the actual problem. For the network traffic classification problem, the overall accuracy is taken as the fitness function. On the Moore dataset and the NISM dataset, the changing curves of the fitness values of the six algorithms in the iteration process are shown in Figs. 5 and 6 respectively.

(2) Comparison of different machine learning algorithms

**Figure 7:**
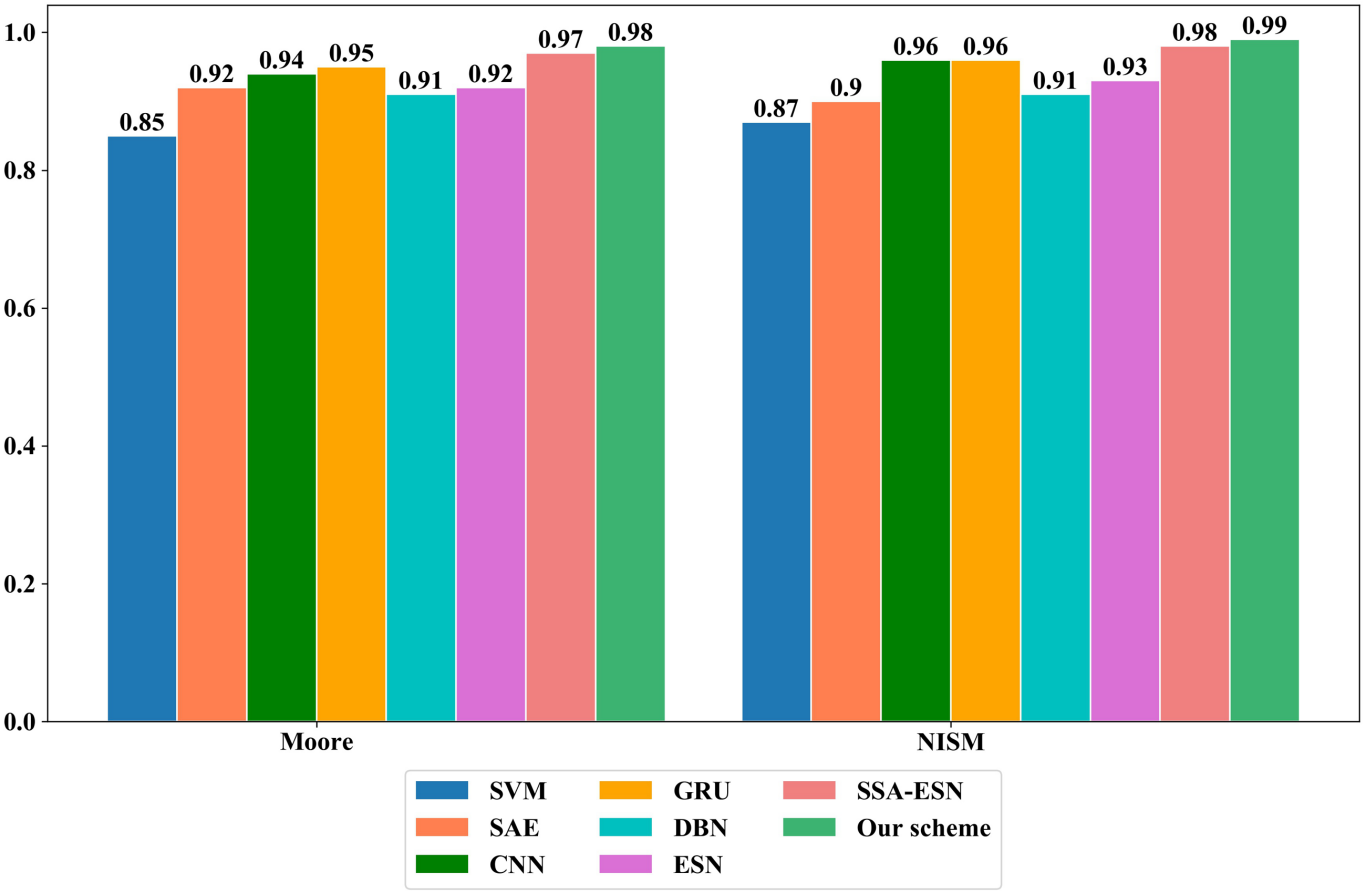
The overall accuracy of different machine learning algorithms on the Moore and NISM datasets.

To verify the effectiveness of the proposed scheme, it is compared with different ML algorithms such as SVM, SAE, CNN, GRU and Deep Belief Networks (DBN) algorithms on Moore dataset and NISM dataset, respectively. On the Moore dataset and the NISM dataset, the parameter values of the comparison algorithms are shown in Tables 5, 6, 7, 8, 9, 10 and 11 and Fig. 7 show the classification results of different algorithms on the two datasets.

**Table 5:** The parameter values of the comparison algorithms.

**Dataset**	**Method**	**Parameter values**
Moore dataset	SVM	RBF kernel, *C* = 1.0, gamma = 0.055
	SAE	Hidden layers=2 (the node number of each layer is 50)
	CNN	Hidden layers=2 (the node number of each layer is 50)
	GRU	Hidden layers=2 (the node number of each layer is 50)
	DBN	Hidden layers=2 (the node number of each layer is 50)
NISM dataset	SVM	RBF kernel, *C* = 1.0, gamma = 0.049
	SAE	Hidden layers=2 (the node number of each layer is 40)
	CNN	Hidden layers=2 (the node number of each layer is 40)
	GRU	Hidden layers=2 (the node number of each layer is 40)
	DBN	Hidden layers=2 (the node number of each layer is 40)

**Table 6:** The class accuracy of different ML algorithms on the Moore dataset.

**Method**	**WWW**	**Mail**	**F-D**	**F-P**	**F-C**	**SERV**	**DB**	**P2P**	**ATT**	**MULT**	**INT**	**Games**
SVM	0.90	0.90	0.93	0.82	0.73	0.88	0.62	0.80	0.82	0.84	0.00	0.00
SAE	0.93	0.97	0.97	1.00	0.83	0.93	0.82	0.79	0.93	0.86	0.83	0.00
CNN	0.94	0.97	0.98	0.95	0.95	0.96	0.94	0.81	0.94	0.89	0.47	1.00
GRU	0.97	0.97	0.97	0.97	0.91	0.99	0.95	0.76	0.99	0.88	0.56	0.00
DBN	0.94	0.92	0.95	0.96	0.80	0.97	0.76	0.79	0.92	0.86	1.00	0.00
ESN	0.92	0.95	0.95	0.90	0.91	0.93	0.92	0.80	0.92	0.86	0.83	0.00
SSA-ESN	0.97	0.97	0.98	0.95	0.93	0.97	0.94	0.89	0.95	0.89	0.90	1.00
Ours	0.99	0.98	1.00	0.95	1.00	1.00	0.99	0.94	0.99	0.91	1.00	1.00

**Table 7:** The class accuracy of different ML algorithms on the NISM dataset.

**Method**	**DNS**	**Ftp**	**Http**	**Telent**	**Lime**	**Local** **forwarding**	**Remote** **forwarding**	**Scp**	**Sftp**	**Shell**	**X11**
SVM	0.90	0.94	0.98	0.97	0.76	1.00	1.00	0.66	0.68	0.91	0.92
SAE	0.90	0.94	0.99	0.93	0.81	1.00	1.00	0.70	0.76	0.93	1.00
CNN	0.95	1.00	1.00	1.00	0.81	1.00	1.00	0.99	0.95	0.97	1.00
GRU	0.85	0.98	0.99	0.96	1.00	0.99	1.00	0.98	0.94	0.97	0.99
DBN	0.91	0.94	0.99	0.99	0.82	1.00	1.00	0.72	0.81	0.94	0.99
ESN	0.89	0.94	0.99	0.94	0.89	1.00	1.00	0.85	0.80	0.94	0.99
SSA-ESN	0.97	1.00	0.99	1.00	0.98	1.00	1.00	0.98	0.98	0.98	1.00
Ours	0.99	1.00	1.00	1.00	0.99	1.00	0.99	0.99	0.99	0.99	1.00

**Table 8:** The class recall rate of different ML algorithms on the Moore dataset.

**Method**	**WWW**	**Mail**	**F-D**	**F-P**	**F-C**	**SERV**	**DB**	**P2P**	**ATT**	**MULT**	**INT**	**Games**
SVM	0.97	0.88	0.92	0.82	0.75	0.94	0.89	0.37	0.81	0.82	0.00	0.00
SAE	0.98	0.92	1.00	0.96	0.90	0.94	0.98	0.71	0.84	0.86	0.50	0.00
CNN	0.99	0.96	1.00	0.93	0.86	0.95	0.95	0.83	0.88	0.83	0.70	0.50
GRU	0.97	0.98	1.00	0.97	0.89	0.94	0.97	0.94	0.83	0.87	0.50	0.00
DBN	0.98	0.92	0.99	0.93	0.87	0.93	0.95	0.70	0.81	0.81	0.20	0.00
ESN	0.97	0.92	0.92	0.93	0.85	0.94	0.95	0.89	0.83	0.81	0.50	0.00
SSA-ESN	1.00	0.98	1.00	0.99	0.95	0.99	0.98	0.94	0.94	0.94	0.80	0.50
Ours	1.00	0.99	1.00	0.99	0.96	0.99	0.98	0.95	0.95	0.95	0.90	0.50

**Table 9:** The class recall rate of different ML algorithms on the NISM dataset.

**Method**	**DNS**	**Ftp**	**Http**	**Telent**	**Lime**	**Local** **forwarding**	**Remote** **forwarding**	**Scp**	**Sftp**	**Shell**	**X11**
SVM	0.72	1.00	0.96	0.93	0.90	0.97	0.98	0.67	0.67	0.92	0.97
SAE	0.79	1.00	0.99	0.93	0.91	0.99	0.98	0.76	0.68	0.97	0.98
CNN	0.78	1.00	0.99	1.00	0.96	0.99	0.98	0.97	0.99	1.00	0.99
GRU	1.00	1.00	0.99	0.98	0.81	0.99	0.98	0.94	0.97	0.99	0.99
DBN	0.80	1.00	1.00	0.93	0.91	0.96	0.99	0.81	0.71	1.00	0.98
ESN	0.80	1.00	1.00	0.93	0.91	0.97	0.98	0.87	0.85	0.95	0.98
SSA-ESN	0.95	1.00	1.00	1.00	0.96	0.99	0.99	0.97	0.97	0.99	0.98
Ours	0.99	1.00	1.00	1.00	0.99	0.99	0.99	0.99	0.99	0.99	0.99

**Table 10:** The class F-measure of different ML algorithms on the Moore dataset.

**Method**	**WWW**	**Mail**	**F-D**	**F-P**	**F-C**	**SERV**	**DB**	**P2P**	**ATT**	**MULT**	**INT**	**Games**
SVM	0.93	0.89	0.92	0.82	0.74	0.94	0.73	0.50	0.81	0.83	0.00	0.00
SAE	0.95	0.94	0.99	0.98	0.86	0.94	0.89	0.75	0.88	0.86	0.62	0.00
CNN	0.97	0.97	0.99	0.94	0.90	0.95	0.94	0.82	0.91	0.86	0.56	0.67
GRU	0.97	0.97	0.99	0.97	0.90	0.97	0.96	0.84	0.90	0.87	0.53	0.00
DBN	0.96	0.92	0.97	0.95	0.83	0.95	0.84	0.74	0.86	0.84	0.33	0.00
ESN	0.96	0.92	0.95	0.90	0.88	0.94	0.94	0.80	0.88	0.86	0.70	0.00
SSA-ESN	0.99	0.97	0.99	0.97	0.97	0.99	0.99	0.94	0.95	0.93	0.90	0.67
Ours	1.00	0.98	1.00	0.97	0.98	0.99	0.99	0.95	0.97	0.93	0.95	0.67

**Table 11:** The class F-measure of different ML algorithms on the NISM dataset.

**Method**	**DNS**	**Ftp**	**Http**	**Telent**	**Lime**	**Local** **forwarding**	**Remote** **forwarding**	**Scp**	**Sftp**	**Shell**	**X11**
SVM	0.80	0.97	0.97	0.95	0.83	0.99	0.99	0.67	0.68	0.91	0.95
SAE	0.84	0.97	0.99	0.93	0.86	0.99	0.99	0.73	0.72	0.95	0.99
CNN	0.86	1.00	1.00	1.00	0.88	0.99	0.99	0.98	0.97	0.98	0.99
GRU	0.92	0.99	0.99	0.97	0.90	0.99	0.99	0.96	0.96	0.98	0.99
DBN	0.85	0.97	0.99	0.96	0.86	0.98	0.99	0.76	0.76	0.97	0.99
ESN	0.80	0.97	0.97	0.92	0.88	0.99	0.99	0.89	0.85	0.95	0.99
SSA-ESN	0.98	0.99	1.00	0.97	0.98	0.99	0.99	0.98	0.99	0.99	0.99
Ours	0.99	1.00	1.00	1.00	1.00	0.99	0.99	0.99	0.99	0.99	0.99

For network traffic classification problems, classification time is also an important indicator. Classification time includes training time and testing time. The training time and testing time of each algorithm on the datasets are shown in Table 12.

**Table 12:** The training time and testing time of each algorithm.

Algorithms	Moore dataset		NISM dataset	
	Training time(s)	Testing time(s)	Training time(s)	Testing time(s)
SVM	35.613	0.2031	89.7042	0.9505
SAE	60.437	1.3562	113.489	2.369
CNN	38.1249	0.2504	56.3493	1.3795
GRU	31.8204	0.4143	52.2412	0.9124
DBN	33.8685	0.4361	52.1904	0.9579
ESN	9.892	0.1998	19.593	0.8237
SSA-ESN	250.078	0.4036	372.6851	0.9601
Ours	233.441	0.3983	345.7114	0.9582

## Discussion

As shown in Figs. 5 and 6, the ASSA initializes the population using the Tent mapping with reverse learning, which promotes the uniform distribution of the initial population and improves the search efficiency. Then, it introduces polynomial operator to maximize the diversification of search domain, which improves the global exploration ability. Finally, the ASSA introduces a dynamic mutation strategy to increase the population diversity at the later stage and avoid local optimum. Therefore, the ASSA has better fitness value and converges faster during the iteration process compared with PSO, FOA, SSA, GA, and GWO.

It can be seen from Tables 6–11 and Fig. 7 that the proposed scheme has obvious advantages over traditional ML methods on the two datasets in the class accuracy, class recall rate, class F-measure and overall accuracy. The reasons for the better classification performance of the proposed scheme are as follows: (1) The ESN processes information by simulating the thinking mode of human brain and has the characteristics such as self-organization, self-learning and self-adaptation. It adopts the reservoir composed of sparsely connected neurons as the hidden layer to perform high-dimensional and non-linear representation of the input data. It only trains the weights from the reservoir to the output layer, which simplifies the training process. (2) The Tent mapping with reverse learning, polynomial operator and dynamic mutation strategy are introduced to improve the SSA, which improves global exploration ability of the algorithm and avoids the algorithm from falling into the local optimum. The ASSA is then used to automatically optimize the hyperparameters of the ESN and can accurately find the optimal hyperparameters of the ESN.

It can be seen from Table 12, due to use ASSA to optimize the hyperparameters of ESN, the training time is longer than other approaches. The trained model is tested on the testing set. The testing time of our method and other approaches is not much different. Especially when the testing time is averaged to each sample, this difference is very small. In addition, our method has obvious advantages over other approaches in terms of per-class metrics and overall accuracy. Therefore, the gap between the classification time of our method and other methods is acceptable.

## Conclusion

We propose a classification method of Internet traffic based on ESN. Firstly, Tent mapping with reverse learning, polynomial operator and dynamic mutation strategy are introduced to improve the SSA. Then, the advanced SSA is used to optimize the hyperparameters of the ESN such as the size of the reservoir and spectral radius. Finally, the optimized ESN is adopted to classify network traffic. We evaluate the performance of the proposed scheme on Moore and NISM datasets, and perform comparison experiments with SVM, SAE, CNN, GRU and DBN algorithms in terms of per-class metrics and overall accuracy, respectively. Experimental results show that our method has advantages in multiple evaluation metrics compared with these traditional ML algorithms and effectively improves the accuracy of network traffic classification. Our method needs further experimental verification before it can be deployed in the Internet environment. In the further, how to improve the real-time performance of the network traffic classification method still needs further research.

## Additional Information and Declarations

### Data Availability

The following information was supplied regarding data availability: The code is available at GitHub: https://github.com/melody213/network. The third-party data is available at: - Moore dataset: https://www.cl.cam.ac.uk/research/srg/netos/projects/archive/nprobe/data/papers/sigmetrics/index.html
- NISM dataset: https://projects.cs.dal.ca/projectx/Download.html
